# Polyphenol Intake in Elderly Patients: A Novel Approach to Counteract Colorectal Cancer Risk?

**DOI:** 10.3390/ijms26062497

**Published:** 2025-03-11

**Authors:** Stefania Fumarola, Laura Cianfruglia, Monia Cecati, Cinzia Giammarchi, Salvatore Vaiasicca, Massimiliano Gasparrini

**Affiliations:** 1 Advanced Technology Center for Aging Research, Istituto di Ricovero e Cura a Carattere Scientifico, Istituto Nazionale di Ricovero e Cura per Anziani (IRCCS-INRCA), 60121 Ancona, Italy; s.fumarola@inrca.it (S.F.); l.cianfruglia@inrca.it (L.C.); 2 Department of Human Sciences and Promotion of the Quality of Life, San Raffaele Roma Open University, 00166 Rome, Italy; monia.cecati@uniroma5.it; 3 Scientific Direction, Istituto di Ricovero e Cura a Carattere Scientifico, Istituto Nazionale di Ricovero e Cura per Anziani (IRCCS-INRCA), 60121 Ancona, Italy; c.giammarchi@inrca.it; 4 Center for Neurobiology of Aging, Istituto di Ricovero e Cura a Carattere Scientifico, Istituto Nazionale di Ricovero e Cura per Anziani (IRCCS-INRCA), 60121 Ancona, Italy; 5 Department of Agriculture, Food and Environmental Sciences, Polytechnic University of Marche, 60131 Ancona, Italy

**Keywords:** polyphenols, colorectal cancer, elderly patients, molecular targets, age-related CRC

## Abstract

Colorectal cancer (CRC) accounts for approximately 10% of all cancers worldwide with an incidence of approximately 60% in patients older than 70 years. In the elderly, the definition of a better therapeutic strategy depends on several factors including the patient’s frailty and comorbidity status, life expectancy, and chemotherapy tolerance. In older patients, adverse drug reactions require a reduction in the dose of treatment, resulting in worse oncologic outcomes. In recent years, an increasing number of studies have focused on the potential effects of polyphenols on human health and their use in cancer therapy. In this comprehensive review, we searched the major databases and summarized experimental data of the most important polyphenols in the CRC chemoprevention, with a focus on the molecular mechanisms involved and the antitumor effects in the elderly population. In vitro and in vivo studies have shown that polyphenols exert chemopreventive activity by modulating cell signaling, resulting in the inhibition of cancer development or progression. However, the efficacy seen in experimental studies has not been confirmed in clinical trials, mainly due to their low bioavailability and non-toxic doses. Further research is needed to increase polyphenol bioavailability and reduce side effects in order to suggest their possible use to increase the efficacy of chemotherapeutic treatment.

## 1. Epidemiology and Risks Factors of Colorectal Cancer

Colorectal cancer (CRC) accounts for approximately 10% of cancer cases globally [1]. After breast and lung cancer, CRC is the third most common cancer in terms of new cases, with nearly 1.9 million cases. However, it ranks second in terms of mortality, with more than 904,000 deaths recorded in 2022 [2]. CRC is the second most common cancer diagnosed in women and the third most common in men. However, women have approximately 25% lower incidence and mortality rates compared to men. Due to advances in diagnostic methods, the global number of newly diagnosed cases of CRC worldwide is projected to increase to 2.5 million by 2035 in developing countries [3]. Europe has the highest age-standardized incidence rate (ASR) of 30.4, followed closely by Oceania at 29.8. North America has a rate of 26.2, while Asia and Latin America have rates of 17.6 and 16.6, respectively. The Eastern Mediterranean region has a rate of 9.1 and Africa has the lowest ASR for CRC at 8.4. The potential causes of CRC, such as environmental and genetic influences, can be divided into modifiable aspects such as alcohol consumption, smoking, obesity, poverty, diet, and unmodifiable factors such as age, gender, family history of CRC, gut bacteria, and genetic predisposition [4].

## 2. Molecular Basis of Colorectal Cancer and Chromosomal Instability

**Figure 1:**
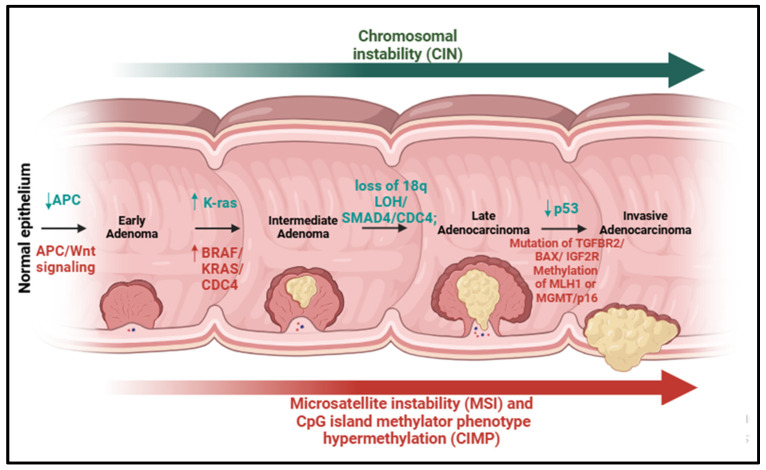
Genetic changes driving advancement of colorectal cancer. Chromosomal instability (CIN), microsatellite instability (MSI), and CpG island hypermethylation (CIMP) are three mechanisms that regulate progression of adenocarcinoma by activating oncogenic mediators and inhibiting suppressor factors. The arrows (↑) and (↓) denotes increasing and decreasing activity, respectively.

Approximately 70% of CRC cases arise spontaneously from the alteration of specific physical characteristics, progressing from adenoma to carcinoma, while 5% are due to genetic factors (such as Lynch syndrome, familial adenomatous polyposis, and MUTYH-associated polyposis), and the remaining 25% have a familial link; a small number of cases are associated with high microsatellite instability and DNA mismatch repair deficiency [5,6]. Chromosomal instability (CIN), microsatellite instability (MSI), and CpG island methylator phenotype hypermethylation are the three pathways that control the sequence of adenocarcinomas (Figure 1).

CIN, known as the major genetic instability in CRC, is characterized by a significant increase in chromosomal gain or loss, which some studies suggest is due to the presence of potential tumor suppressor genes or oncogenes [7]. In particular, the inactivation of chromosome 18q LOH, APC, and TP53 and the activation of KRAS and BRAF account for 85% of adenocarcinoma mutations. This allows the modified cells to multiply and eventually turn healthy cells into cancerous ones [7]. Another type of genomic instability in CRC is MSI, a hallmark of cancer cells. However, in the majority of sporadic CRCs, the underlying mechanism for CIN is still evolving and MSI accounts for only 15–20% of all CRC cases. TGF-βR2 was the most commonly mutated locus, and polyadenine tract instability of TGF-βR2 occurs in approximately 85% of MSI-H colorectal cancers. In addition, Bax, the other commonly altered gene, was found to have frameshift mutations within the polyguanine region in almost 50% of MSI-H CRCs, leading to its inactivation and a blockade of apoptosis [8]. MSI is rarely observed in polyps, except in cases of Lynch syndrome: in this case, CRC often expresses MSI as a result of germline mutations in one of the mismatch repair (MMR) genes (MLH1, MSH2, MSH6, and PMS2). The third mechanism of CRC progression is related to epigenetic instability, characterized by hypermethylation of CpG island-containing loci, typically associated with global DNA hypomethylation. Changes in the methylation patterns can affect almost all signaling pathways, such as TP53, TGFβ/SMAD, and Wnt, which are involved in cell cycle control, transcriptional regulation, DNA stability, apoptosis, cell adhesion, angiogenesis, cell invasion, and metastasis [9].

## 3. Inflammatory Bowel Disease and CRC

Although patients with inflammatory bowel disease (IBD) are at increased risk of developing CRC, the overall incidence of IBD-associated CRC has decreased significantly in Western countries in recent decades, probably due to advances in medical therapies and the introduction of endoscopic screening/surveillance [10]. Previous studies have shown that the risk of CRC in IBD increases with long-term, family history of CRC, coexisting primary sclerosing cholangitis, extent of colitis, and degree of inflammation [11], although the pathogenesis of this condition is not well understood. IBD-associated CRC may result from sequential episodes of genomic, histological, and genetic changes, as in sporadic CRC. It may also be associated with immune responses promoted by mucosal inflammatory mediators, gut microbiota, and oxidative stress. Other important risk factors include early-onset IBD in younger individuals, colonic inflammation, the severity of inflammation, backwash ileitis, the presence of strictures, and post-inflammatory polyps [12]. It remains unclear whether the increased risk of CRC is due to a higher incidence of dysplastic polyps, which are difficult to distinguish from benign post-inflammatory polyps during surveillance colonoscopies, or because post-inflammatory polyps indicate more severe inflammation [13]. In order to reduce the risk of CRC, surveillance strategies for patients with IBD were recommended based on risk stratification, which accurately differentiates between high-risk and low-risk individuals [14]: in this sense, an individualized approach is essential to achieving optimal disease control, including new therapeutic targets and interventions that could reduce the risk of carcinogenesis.

## 4. Management of CRC

There are five stages of CRC: the initial stage (stage 0) indicates the presence of atypical cells in the mucosal layer of the colon wall. In stage I, the tumor invades the submucosal layer of the colon or rectum, while in stage II, the cancer has spread through the wall to the serosa but has not reached adjacent organs. Stage III refers to the presence of cancer in the mucosa, submucosa, and serosa and the involvement of adjacent lymph nodes. Stage IV represents the most severe form of CRC, in which the cancer metastasizes and spreads to different parts of the body [15]. Improvements in (i) national screening programs, (ii) availability of colonoscopy, and (iii) lifestyle and diet have significantly reduced the number of new CRC cases, particularly in highly developed countries [16]. Treatment of CRC is determined by factors such as tumor location, stage, and the patient’s overall status. Surgery is the preferred treatment for stage I or II CRC, while stage III and metastatic CRC may require systemic chemotherapy or a combination of targeted biological drugs. Alternatively, treatment for rectal cancer includes surgery or radiotherapy for stage I, while stage II and III patients may undergo short-term chemoradiotherapy followed by surgical resection and adjuvant chemotherapy [17].

## 5. CRC in Elderly Patients

**Figure 2:**
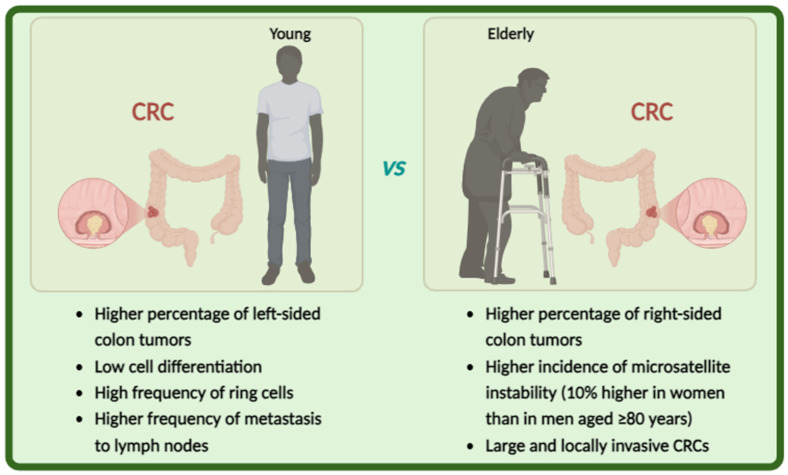
Potential mechanisms implicated in age-related carcinogenesis of CRC.

CRC is more common in older people than in younger ones. Approximately 60% of individuals diagnosed with CRC are over 70 years of age and 43% are over 75 years old [18]. In the older population, aggressive treatments such as surgery or proposed adjuvant or neoadjuvant therapy are not recommended [19]. Viewing aging as a gradual and individualized decline in the functional capacity of various organs, the approach to CRC management should focus on assessing physiological and non-chronological age, comorbidities, life expectancy, and ability to tolerate treatment. Several studies have shown that the characteristics of CRC in older patients differ significantly from those in younger subjects (Figure 2).

Approximately 20% of early-onset CRC can be attributed to a familial syndrome, while the majority are typically microsatellite-stable tumors. Young adults with early-onset CRC have delayed diagnosis, low cell differentiation, and a high frequency of ring cells, often affecting the left side of the colon. In contrast, older patients with colon cancer have a higher percentage of right-sided colon tumors and a higher incidence of microsatellite instability than younger patients [20]. This incidence is about 10% higher in women than in men aged ≥80 years. In addition, older patients tend to have large and locally invasive CRCs, but the frequency of lymph node metastasis is lower than in younger patients [21]. Tumors with mismatch repair deficiency and MSI are more common in older patients with CRC, occurring in 36% of patients aged ≥85 years, with a particularly high frequency in women [22]. In right-handed, high-MSI CRCs developed in older women, the hMLH1 gene promoter is often methylated and its protein expression is silenced [23].

Because of the clinical condition of older people, there is a risk of inadequate treatment, resulting in a poorer outcome in terms of cancer regression. Overly forceful and invasive procedures can have harmful and fatal consequences. Because of these factors, older individuals classified as “frail subjects” may be suitable candidates for palliative care, to improve their quality of life. This treatment is particularly difficult for older people to accept, as it involves balancing their quality of life with the chance of eliminating the tumor. However, when the patient is extremely debilitated, palliative care is an effective tool to alleviate pain, maintain autonomy, and enhance overall well-being [24].

## 6. Polyphenols and Their Role in CRC Prevention

**Figure 3:**
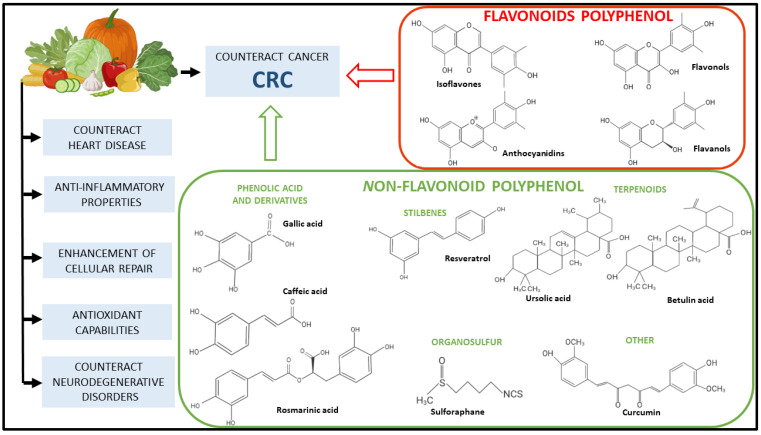
Classification of polyphenols related to CRC. Principal properties of fruits and vegetables related to CRC are reported. Chemical structures of flavonoid and non-flavonoid polyphenols are also represented.

Polyphenols are naturally occurring bioactive substances present in plant-based foods. Due to their prevalence in vegetables and herbs, in recent years, an increasing number of studies have focused on the potential effects of polyphenols on human health. Another factor that affects their popularity is their minimal side effects and their accessibility and affordability compared to their synthetic drugs [25,26]. In recent years, there has been consistent progress in recognizing plants’ bioactive secondary metabolites, such as phenolic compounds, and in the understanding of their mechanisms of action, in order to clarify their health benefits [27]. Several studies have observed a positive association between phytochemicals and CRC prevention, with a focus on exploring their anti-cancer properties in different CRC models [28,29] (Figure 3).

## 7. Research Methodology

**Figure 4:**
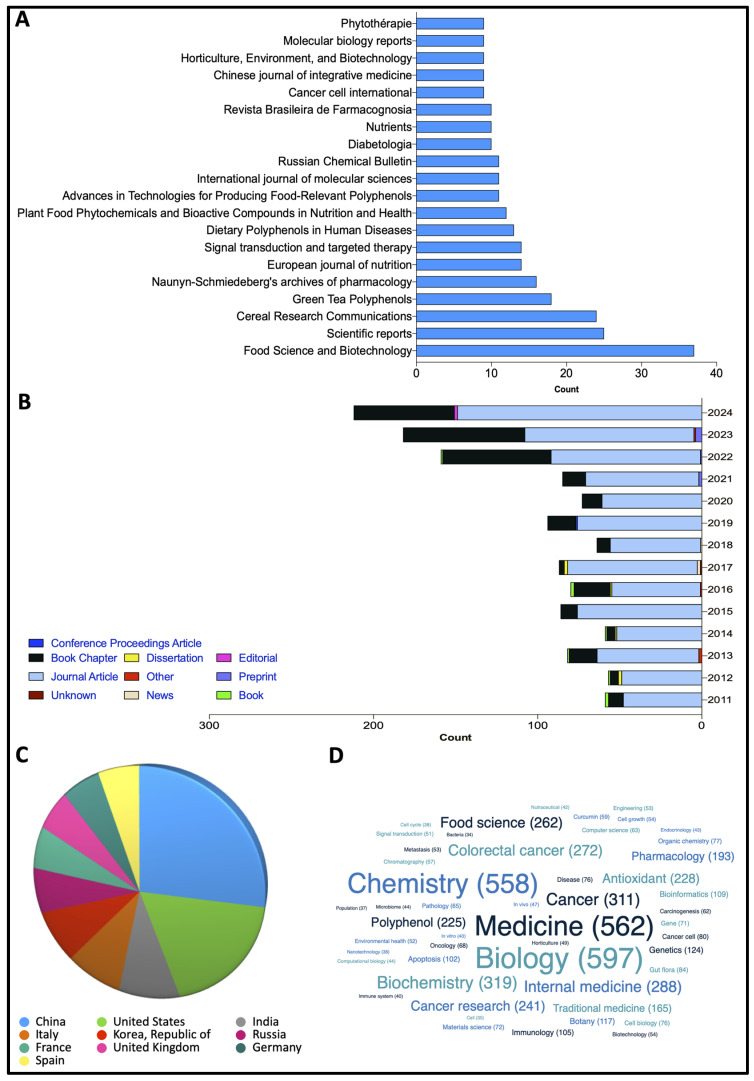
Comprehensive overview conducted on lens.org libraries showing highest level of consistency in publishing throughout researched years (2011–2024). (**A**) Distribution of publication per journal. (**B**) Distribution of publications per year and document type from. (**C**) Number of publications per country. (**D**) Number of publications per application field.

The scientific literature review includes the results of searching using the following keywords, “polyphenols”, “CRC”, “colorectal cancer”; “elderly patients and CRC”; “CRC molecular targets”; “age-related CRC”; “phytochemical”, in major databases such as Web of Science, Scopus, Elsevier, Springer, and Google Scholar from 2011 to 2024 for preclinical and clinical studies. Special attention was paid to the molecular mechanisms involved in the protective effects against oxidative and inflammatory stress and their effect on CRC carcinogenesis in the older population. A review of the scientific papers obtained from 2011 to 2024 was also conducted on the lens.org website, using the search terms “CRC and Polyphenols”. The results of this search are illustrated in Figure 4, which shows the number of papers, field applications, and publications per country and per journal. All papers used were published and peer-reviewed by several experts in the field.

## 8. Polyphenols for CRC Prevention: Preclinical Studies

### 8.1. Flavonoids

#### 8.1.1. Quercetin

Flavonoids are the most abundant group in the polyphenolic classification which is divided into six classes: flavonols, anthocyanins, isofalvons, flavanols, flavanones, and flavons. They have in common the presence of two benzene rings that can be linked with a three carbon chain, with a general backbone structure of C6-C3-C6 [30]. One of the flavonoids with important anti-cancer properties is quercetin (C_15_H_10_O_7_). It is a flavonol that is present in several natural compounds such as apple, tea, honey, tomato, strawberry, and, in particular, red onion [31]. Quercetin and its derivatives have shown anti-cancer effects in colon cancer by inhibiting cell proliferation and cell cycle arrest, inducing apoptosis or autophagy, and reducing tumor growth. In 2012, Kim et al. demonstrated that quercetin induced apoptosis in the HCT-116 Xenograft model by reducing Hypoxia inducible factor (HIF)-1, a crucial dimeric protein complex involved in the response to low oxygen concentrations and thus in anaerobic metabolism and the regulation of homeostatic processes. In the same year, Bulzomi et al. observed in a colorectal adenocarcinoma cell line (DLD-1) the increase in three molecular targets (caspase-3, c-PARP, and p-38MPK) involved in the apoptosis process, after quercetin treatment [32]. Similar effects were observed in other CRC cells such as CT-26, Caco-2, SW-620, and HT-29 [33]. In particular, the increase in molecular targets such as Bax, p53, caspase-3, and -9 and the decrease in bcl-2, p-Akt followed by the arrest of cell cycle in G0/G1 phase were highlighted [34,35,36]. In addition to its anti-cancer activities, quercetin exerts an anti-inflammatory function: in Caco-2 cells, Han et al. observed the decrease in the expression levels of several genes involved in pro-inflammatory response, such as TLR4, NF-kb, TNF-α, COX-2, and IL-6 [37]. On the other hand, a quercetin derivate (synthesized quercetin 5,3-dihydroxy-3,7,4-triethoxyflavone (TEF)) induced, in HTC-116 cells, oxidative stress, as demonstrated by the increase in ROS level followed by the promotion of the apoptotic process [38]. Despite this, clinical trials conducted in patient populations with documented CRC pathology do not provide a strong support for the beneficial effects of quercetin. Only one clinical trial conducted by Cruz-Correa and colleagues in 2006 supports the role of quercetin as a chemopreventive agent. This study involved five patients with previous colectomy and familial adenomatous polyposis. After a supplementation regimen with 60 mg of quercetin and 1.44 g of curcumin per day for 6 months, a reduction in the number and size of adenomatous polyps was observed [39].

Initial data appear promising; however, population-based statistical studies on quercetin consumption and the onset of colorectal cancer are limited and inconclusive. Bobe et al. found an inverse association among isorhamnetin, kaempferol, and quercetin intake and serum interleukin-6 levels, as well as an increased risk of cancer development and recurrence of advanced adenomas. However, these findings may not be relevant to the general population, as they often suggest flavonol consumption levels that exceed those typically consumed in the United States (30 mg/day in our study versus 8–12 mg/day in the general U.S. population) and most participants had a healthy lifestyle [40]. However, these data suggest that translating quercetin targets from model organisms to patients with CRC pathology will provide promising new therapeutic avenues in this field.

#### 8.1.2. Kaempferol

Kaempferol is the major flavonoid aglycone found in many natural products such as apples and grapes and in plants such as green tea. The main source of this flavonol is represented by blackcurrants [41]. Kaempferol and its glycosylated derivatives were demonstrated to have several protective effects including neuroprotective, anti-inflammatory, antioxidant, antimicrobial, and anti-cancer activities [42]. Epidemiological studies have shown that a higher intake of kaempferol is associated with reduced cancer rates in various organs such as the skin, liver, colon, ovary, pancreas, stomach, and bladder [43]. In this context, researchers have become increasingly interested in studying kaempferol and investigating its potential application in cancer therapy [43]. Kaempferol was reported to have cytotoxic effects by inducing apoptosis in various human CRC cell models, including HCT116, HT-29, HCT-15, LS174-R colon, and SW480 cells [44,45]. In particular, in HT-29 cells after treatment, the researcher showed a significant downregulation of PI3K/Akt and ERK-1/2 pathways by inhibiting IGF-IR and ErbB3 signaling [46] with a consequent increase in c-Caspase-3, -7, -9, PARP, Bik, and Bad [45]. In addition, kaempferol can block both G1 and G2 phases of the cell cycle, reducing the levels of CDK2, CDK4, Cdc25c, cyclin B1, cyclins D1, cyclin E, and cyclin A [47]: this property could be used to prevent the proliferation of colon cancer cells. The effect of Kaempferol on the epigenetic aspect was also observed with a hyperacetylation of the histone complex H3. A similar situation was visualized in SW-480 and SW-620 cell models: in both cases, kaempferol treatment promotes apoptosis processes through DNA fragmentation and the activation of caspase-3, -8, -9, and -10 [48,49].

#### 8.1.3. Anthocyanin

Another important class of compounds in the flavonoids group are anthocyanins (C_15_H_11_O_6_CI). These characteristic pigments are present in several sources such as strawberry, blueberry, grapes, purple cauliflower and, in particular, purple corn [50]. In vitro studies have demonstrated their anti-toxic and anti-carcinogenic effects mainly due to their ability to scavenge ROS by chelating metals and by directly binding to proteins, such as active enzymes like superoxide dismutase [51]. The ability of anthocyanins to chelate metals, such as alumin, copper, iron, cadmium, and zinc, is determined by the 3′,4′-O-dihydroxyl group in the B-ring of the flavonoid skeleton [52]. They are also involved in reducing cellular proliferation by modulating signaling through the Wnt/b-catenin and NF-kB pathways [53,54]. The potential role of anthocyanins in the prevention and suppression of CRC proliferation was also reported in various studies through the induction of caspase 3 and 7, the upregulation of p21, and the suppression of TNFα and the NF-kB expression [55,56,57]; interestingly, the anti-proliferative effect exerted by anthocyanin selectively inhibits the growth of colon cancer cells with respect to breast cancer, characterized by low cytotoxic levels [58].

The administration of anthocyanin to colon cancer HCT-116 cells suppressed the migration and invasion of cancer cells through the reduction in p38MAPK, PI3K/Akt, MMP2, and MMP-9 [59]. In addition, Wang et al. observed the demethylation of the tumor suppression genes DNMT1 and DNMT3B in the same cells. These are two important inhibitors that are being developed as potential cancer therapeutics, as they can lead to demethylation and reactivation of the silenced genes [60]. In addition, Mazewski and colleagues observed the induction of apoptosis with the decrease in cIAP-2, Survivin, and XIAP and cell cycle arrest in the G1 phase in HCT-116 cells [61]. Moreover, some studies on Caco-2 and HT-29 cell lines have reported the induction of apoptosis and cell cycle arrest after anthocyanin administration by increasing caspase-3 [54,62].

#### 8.1.4. Genistein

Genistein, also known as 4,5,7-trihydroxyisoflavone, is a natural isoflavone produced as a primary intermediate in the synthesis of more advanced isoflavonoids, which play a crucial role in plant–microbe interaction. The distribution of these isoflavonoids is restricted to the Leguminosae subfamily, in particular, soy, green lentil, and lupine. Genistein exerts anti-invasive and anti-proliferative effects on human CRC cells, resulting in the inhibition of cell proliferation and apoptosis [63,64]. These effects are attributed to its antioxidant effects, the inhibition of tyrosine kinases, cell cycle arrest, estrogen receptor blockage, the induction of differentiation and apoptosis, and the modulation of signaling transduction pathways, such as Wnt/β-catenin and PI3K/Akt [65,66]. The effect of genistein treatment on HT-29 evoked the induction of epigenetic modification by observing a decrease in histone deacetylase 1 (HDAC1) [67]. In the same cellular model, the inhibition of cell proliferation was observed by analyzing the decrease in β-catenin and p-p38 MAPK. Furthermore, treatment with genistein induced apoptosis, as shown by the increase in Bax and the decrease in Bcl-2 and NF-kB in HT-29 and LoVo cells [68]. Genistein has received more attention because, together with its derivates, it exerts its action in various signaling pathways of CRC such as Wnt/β-catenin and NF-kB signaling pathways. The suppression of cell growth was observed in several colorectal cancer cells [69]: the inhibition of cell proliferation was observed by blocking the cell cycle at the G0/G1 [70] and G2/M [71,72] phases with a significant decrease in cyclin B1 and serine/threonine-protein kinase 2 (Chk2) [73]. At the same time, an increase in ATM/p53 and DNA damaging inducible gene 45α (GADD45α) was also demonstrated [69].

#### 8.1.5. Epigallocatechin-3-Gallate

Another important polyphenol is Epigallocatechin-3-gallate or EGCG (C_22_H_18_O_11_), which is produced via the naringenin-chalcone–naringenin-dihydrokaempferol pathway. EGCG is a flavanol and it is mainly present in green tea but also in apple peel, onions, hazelnuts, and carob powder. EGCG possesses various biological activities, including anti-cancer effects as investigated in various in vitro and in vivo studies [74]. Recently, a lot of research has been conducted on the effects of EGCG on colon cancer by modulating numerous signaling pathways, such as Wnt/β-catenin [75] and PI3K/Akt [76]. For example, after the treatment of different human colon cancer cell lines (HCT-116, HT-29, LoVo, and Caco-2), the block of cell proliferation with the induction of the apoptosis state was observed with (i) the increase in p53 [77], p-Erk1/2, p-JNK1/2, and p-p38MAPK [78] and (ii) the decrease in Erk1/2 and NF-kb [79]. It was also observed that EGCG is involved in epigenetic modulation through the reduction in DNMT3A and HDAC3, which are two crucial proteins involved in methylation balance and their reduction is related to cancer proliferation [80]. Furthermore, the same treatment caused a decrease in RXRa, b-catenin, and cyclin D1, which are involved in the cell cycle and thus in tumor proliferation [81]. However, few reports have aimed to evaluate the effects of EGCG and its role in primary and metastatic colon cancer cell lines.

### 8.2. Non-Flavonoid Polyphenol

#### 8.2.1. Resveratrol

Resveratrol (C_14_H_12_O_3_) is a polyphenolic phytoalexin stilbene found mainly in red grapes, but also in berries, plums, and peanuts. Resveratrol exists as both cis- aand trans-isomer: the trans-isomer is the most stable form and also the most extensively studied [82]. Resveratrol is able to bind to various organic compounds, including hormones and enzymes that cross the cell membrane, therefore enhancing multiple biological activities by triggering and activating different signaling pathways. The Phytoalexin contained in resveratrol acts as a natural inhibitor of cell proliferation, reducing tumor growth in animal models [83]. Several ancient medical systems, such “Darakchasava”, an Ayurvedic herb, have used resveratrol for health benefits [84]. However, since its discovery in 1940 [85], the first real interest in this compound occurred in 1992, when resveratrol was proposed to explain some of the cardioprotective effects of red wine [86]. Subsequently, Jang and colleagues observed the ability of resveratrol to inhibit carcinogenesis at multiple stages [87]. This was the starting point for a detailed investigation of the chemopreventive and therapeutic effects of resveratrol in a wide range of cancer models, including CRC.

It remains unclear how resveratrol exerts its beneficial effects: it appears to have intrinsic antioxidant and anti-inflammatory capacities, and both mechanisms contribute to an overall reduction in oxidative stress. As widely reported in the literature, the resveratrol exerts anti-inflammatory effects, as observed in Caco-2 cells through the reduction in pro-inflammatory markers, such as iNOs, TLR-4, p-IkB, and NF-kB [88]. In the same cells and in the HCT-116 model, resveratrol treatment caused cell cycle arrest at the G1/S phase and induced the activation of caspase-7 and -9 [89]. In addition, it was observed that the inhibition of cell proliferation was strictly related to the reduction in PI3K/Akt, Wnt/b-catenin, and cyclin D1 expressions, as demonstrated in the HCT 116 cell line [90]. Another important mechanism highlighted in HCT-116 and SW-480 cell models was the suppression of cell migration and invasion, mediated by the inhibition of MMP-7 [91], MMP-9, and C-X-C chemokine receptor type 4 (CXCR4) [92]. Resveratrol induced tumor cell death by modulating different signaling pathways through Fas and Fas-ligand (FasL) levels [93]. Alternative mechanisms of Fas-independent cell death were also proposed [94]. In SW-620 and LoVo cells, resveratrol treatment induced apoptosis through the upregulation of pro-apoptotic proteins such as Bok, Bak1, Bik, Bad, Bax, Noxa, Apaf1, and p53 and at the same time, the downregulation of anti-apoptotic proteins such as Bcl-2 and Bcl-xL [95,96]. Previous studies have also shown that resveratrol reduces VEGF expression at the mRNA level by disrupting HIF-1α and reducing Akt and MAPK levels, thereby mitigating cell proliferation, migration, and invasion [97].

Park et al. demonstrated that the analog of resveratrol and bakuchiol induced TRAIL-associated apoptosis by the upregulation of TRAIL receptors, DR4, DR5, caspase-3, -8 and -9, and PARP in HCT-116 [98]. Furthermore, this compound induced cell cycle arrest in the G1 [89], S [99], and G2/M [100] phases, thereby suppressing the progression of CRC cells. Several studies have shown the synergistic effect of resveratrol with other natural compounds or chemopreventive drugs on human cells model of CRC [76,101,102]. Reddivari et al. demonstrated the synergistic effect between resveratrol and grape seed, observing the inhibition of cell proliferation through the downregulation of Wnt/β-catenin, c-MYC, and cyclin D1 and the induction of apoptosis through the activation of p53, cyto-c, Bax/Bcl-2, and c-PARP [102].

Sporadic CRC is caused by the substitution of glycine by aspartic acid at residue 12 in the KRAS gene. Resveratrol inhibits Kras expression in vitro and in vivo through the upregulation of miR-96 in CRC and pancreatic cancer cell lines and in the Krasmut mouse model [103,104]. It also upregulates and downregulates the expression of tumor suppressor miRNA and onco-miRNA, respectively. Specifically, resveratrol suppressed CRC by precisely upregulating miR-34c expression, downregulating MDR-1 gene expression via the PI3K/Akt pathway, and negatively affecting IL-6-triggered CRC progression [105]. In light of these findings, cancer research is now focusing on increasing clinical trials for the treatment of chronic diseases. However, the use of resveratrol in clinical practice is limited, with only two phase I clinical trials. TrialNCT00433576 “www://clinicaltrials.gov (accessed on 9 December 2024)” is investigating the side effects and ideal dosage of resveratrol for treating patients who have had colorectal cancer surgically removed. Resveratrol may prevent cancer cell growth by inhibiting certain enzymes required for cell multiplication. The NCT00256334 trial TrialNCT00433576 “www://clinicaltrials.gov (accessed on 9 December 2024)” was completed and showed that low doses of resveratrol, when combined with other bioactive elements, can block the Wnt pathway in vivo, with this effect being restricted to normal colon mucosa.

#### 8.2.2. Phenolic Acid and Derivates

Phenolic acids have two distinct carbon structures, hydroxycinnamic acid and hydroxybenzoic acid [106]. Hydroxybenzoic acids are generated from benzoic acid and share a common C6-C1 structure [107]. While the chemical structure of hydroxycinnamic is composed by two carbon chains (C6-C3) attached to the phenolic ring [108].

This class of compounds is available in a soluble form, mainly Gallic acid, Rosmarinic acid for the hydroxybenzoic acids, and Caffeic acid for the hydroxycinnamic acids.

Gallic acid (GA) is a 3,4,5-trihydroxybenzoic acid (C_7_H_6_O_5_), found mainly in walnuts, but also in blueberries, apples, flax plant, and green tea [109]. This compound exhibits strong antioxidant activity [110,111] and has anti-cancer, anti-inflammatory, antimicrobial, and anti-fungal properties [112]. GA is able to inhibit cell proliferation by suppressing Wnt/β-catenin signaling [113] and inhibiting the transcription factors AP-1, NF-kb, STAT1, and OCT-1 [54] in HCT-116, HCT-15, and Caco-2 cell models. Moreover, in HCT-15 colon cancer cell lines, GA exhibited an anti-cancerous effect inducing ROS-dependent apoptosis and early events related to apoptosis, such as lipid layer breakage and a reduction in MPP [114]. Furthermore, the treatment promoted cell cycle arrest at the G0/G1 phase by decreasing cyclin D1 levels and induced apoptosis by activating caspase-3 expression [54].

Rosmarinic acid (RA) (C_18_H_16_O_8_) is an ester of Caffeic acid and 3,4-dihydroxyphenyllactic acid and is found in basil, marjoram, oregano, melissa, peppermint, thyme, and rosemary [115]. The anti-cancer effect of this compound was observed in COLO-205 cells, where it activated apoptosis by increasing the expression of two molecules involved in the regulation of cell death, which are Fas and FasL, and the activation of caspase-8, Bid, Bax, and Bad. In addition, a reduction in MMP and an increase in cytochrome c levels that activate caspase-9 and -3 was observed, determining cPARP and DNA fragmentation [116].

Finally, Caffeic acid (C_9_H_18_O_4_) is mainly found in coffee and to a lesser extent in olive oil, grains, and vegetables [117]. Its anti-cancer properties were elucidated in different cell models of CRC (HCT-15, HCT-116 and SW-480), where it induced a cell cycle arrest at the sub G1 phase [118] with a reduction in NF-kb, p-Akt, mTor, Erk1/2, and PCN expression [119]. Furthermore, the treatment induced apoptosis activation, as observed by the increase in ROS and c-PARP levels and the decrease in MMP [120].

#### 8.2.3. Curcumin

Curcumin, (1,7-bis(4-hydroxy-3-methoxyphenyl)-1,6-heptadiene-3,5-dione) (C_21_H_20_O_6_), is the main active component of the spice turmeric (Curcuma longa). Similarly to other polyphenols, curcumin has antioxidant, anti-inflammatory, antibacterial, anti-fungal, antiproliferative, and antitumor activities [121,122].

Curcumin is known to control several signaling pathways that are crucial for the initiation, proliferation, and progression of colon cancer. For instance, curcumin treatment in SW-480 CR cells induced the inhibition of cell proliferation, suppressed the invasion, and reduced the drug resistance, inducing epigenetic modifications. The inhibition of cell proliferation was characterized by the decrease in mTORC1, followed by the increase in p-Erk1/2 and p-AMPKα1 [123]. The suppression of the invasion was observed with a rise in AMPK and a decrease in p65 NF-kB, uPA, and MMP9 [124]. Noratto et al. and Gandly et al. also observed an important induction of epigenetic modifications in HT-29 and SW-480 colon cancer cells, involving a reduction in specific miRNAs, such as miR-20a, miR-27a, and miR-17-5p [125,126]. Interestingly, in the same cells, curcumin analogs alone or in association with other compounds exerted a similar effect, inhibiting cell proliferation with the reduction in GSK-3β [127]. Curcumin treatment could also cause the arrest of the cellular cycle in the G0/G1 phase [128] with a decrease in cyclin D1 and in the meantime the promotion of the apoptotic mechanism with an increase in caspase-3, -8, and -9 [127] and the Bax/Bcl-2 ratio [129]. The effects of curcumin in different colon cancer cell lines (HCT116, HT29, HCT15, Sw480, and Caco-2) were similar: (i) the decrease in pro-protein convertase activity [130], (ii) the induction of apoptosis [131], and (iii) the epigenetic modifications [132], leading to the inhibition of cell growth and a reduction in colony formation [133]. A consistent amount of data supports the role of autophagy in the early stages of CRC development. Specifically, curcumin treatment stimulated autophagosome, increasing the expression of Lamp1, Hsp70 [134], the TFEB lysosome pathway, and LC3-II and decreasing the expression of p62, Akt, and mTOR [135]. Furthermore, the curcumin analog in HT-29 cells caused a decrease in angiogenesis through the increase in VEGF, HIF-1α, STAT-3 and COX-2 [136], the induction of epigenetic modifications, and consequently the increase in therapeutic efficacy through the reduction in miR-21 [137,138]. Curcumin analog treatment in HCT-116 cells arrested proliferation by increasing TNF-α and NF-kB activation [139] and stimulated apoptosis by increasing caspase-3, -7 and -9, cyto-c, c-PARP, ROS, and JNK [139,140,141]. In addition, the curcumin analog promoted ER stress in HTC-116 cells through the increase in CHOP, ATF6, XBP1, GRP78, and HERPUD1 and also promoted the induction of autophagy through the formation of autosome and the increase in LC3-I to LC3-II conversion [141]. Interestingly, results have been reported by several researchers: the concomitant presence of curcumin plus 5-fluorouracil (5-FU) induced in HCT-116, SW-480, and SW-620 cell lines, the suppression of metastatic ability through the decrease in NF-kB, TGF-b and p-Smad2 [142], and the induction of apoptosis with the increase in Bax and cyto-c and the decrease in Bcl-2 [128,143,144].

Recently, it was also suggested that curcumin may influence the gut microbiota, including its abundance, diversity, and composition [145]. Microbiota dysbiosis is most likely involved in the development of CRC. Curcumin administration was shown to increase the presence of high levels of butyrate-producing bacteria, Clostridium clusters IV and XIVa. These two clusters of Clostridium could produce butyrate and induce mucosal Treg cells; curcumin may ameliorate Treg-associated inflammation by promoting the production of these bacteria [146]. However, high doses of curcumin may result to be cytotoxic via histone modification and the modulation of gene expression [147]. Recently, an increasing number of clinical trials with curcumin have been promoted. For CRC, one of the first trials was a phase IIA clinical trial, designed to evaluate whether curcumin treatment could reduce the pro-cancerogenic eicosanoids prostaglandin E2 (PGE2) and 5-hydroxyheicosatetraenoic acid (5-HETE), prevent the formation of aberrant crypt foci (ACF), and inhibit proliferation in normal mucosa [148]. In this study, a 40%reduction in the number of ACF was observed after 30 days of oral curcumin (2 g or 4 g) at a dose of 4g [148]. Another clinical trial was conducted to evaluate the effect of curcumin on the size and number of polyps. Unfortunately, this study did not report any significant differences [149]. Cruz-Correa and colleagues investigated the synergic effect of curcumin with other inhibitors. In this study, five patients with familial adenomatous polyposis (FAP) were enrolled and given 480 mg of curcumin and 20 mg of quercetin orally 3 times a day. Interestingly, after 6 months of treatment with curcumin and quercetin, all patients had a significant reduction in the number and size of polyps, demonstrating the chemopreventive effect of curcumin in preventing the formation of pre-cancerous polyps in the colon [39].

### 8.3. Terpenoids

Another class of natural compounds with a possible role in anti-cancer activity and, in particular, in colon cancer are terpenoids (C_5_H_8_)n, where “n” refers to the number of isoprene units. Terpenoids are classified as mono, di, oligo, and polyterpenes. Several in vitro and in vivo studies, as well as human epidemiological trials, suggest an antiproliferative effect of terpenoids against various types of cancer [150]. Triterpenoids are isopentenyl pyrophosphate metabolites that exist in several forms, including free terpenoids, saponins, and phytosterols. The main representatives are betulinic and ursolic acid.

#### 8.3.1. Betulinic Acid

Betulinic acid, a pentacyclic triterpene isolated from the bark of Betula pubescens [151], is widely distributed in a variety of plants, especially birch, eucalyptus, and sycamore trees [151]. The anti-cancer effect of betulinic acid was reported in HT-29 colon cancer cells, where it inhibited proliferation and migration via apoptosis induction [152]. In addition, betulinic acid treatment in SW480 and RKO colon cancer cells downregulated pro-oncogenic genes, such as Sp1, Sp3, and Sp4 [153]. Dutta et al. reported that betulinic acid analog induced autophagy in HT-29 cells by increasing Beclin1, Atg 3, Atg 5, Atg 7, and Atg 5–12 and decreasing p62 [154]. Furthermore, treatment with betulinic acid derivates induced apoptosis through an increase in ROS levels and an increase in DNA fragmentation [152].

#### 8.3.2. Ursolic Acid

Ursolic acid (UA) is a natural pentacyclic triterpenoid carboxylic acid with the ability to inhibit the growth of HT-29 cells by decreasing SHH, p-STAT3, pAkt, p-p70S6K [155], PCNA p-Erk, p-JNK, and p-p38MAPK. Furthermore, in the same cellular model, UA treatment forced cell cycle arrest at the G1/S phase and the induction of apoptosis with a decrease in the Bax/Bcl2 ratio [156]. In SW480 cells, ursolic acid treatment decreased the expression of p-PI3K/Akt, p-Erk, p-mTor, COX-2, PGE-2 and NF-kB, arrested CRC cell proliferation, and suppressed migration through the increase in c-PARP, caspase-3 and -9, and cyto-c and the reduction in MMP-9 [157]. Ursolic acid also exerted anti-inflammatory effects by inhibiting the activation of COX-2, PGE2, and NF-kB [158]. In addition, anti-invasive and anti-angiogenesis activity were found in CRC after UA treatment with the downregulation of MMP-2, VEGF, ICAM-1, and bFGF expression and the upregulation of CDH1 [60,157,159].

### 8.4. Organosulfur

**Figure 5:**
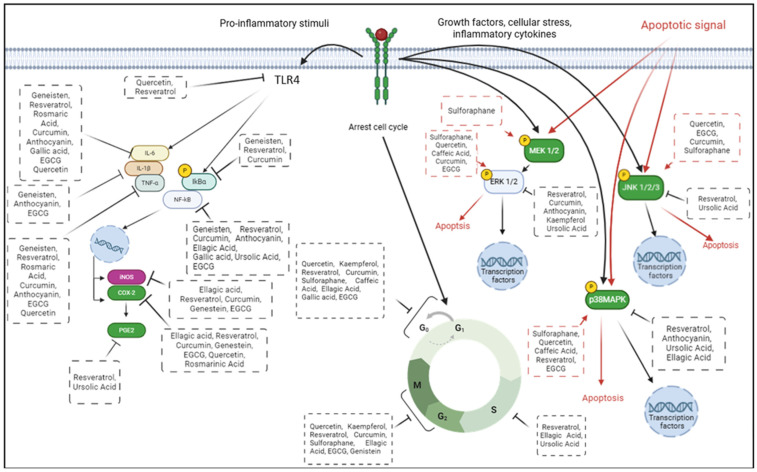
The main molecular mechanisms of colorectal cancer carcinogenesis controlled by different polyphenols.

The organosulfur compounds, mainly sulforaphane (C_6_H_11_NOS_2_), are the last class of polyphenols to be investigated for their anti-cancer activity in CRC models. Sulforaphane is an isothiocyanate found mainly in cruciferous vegetables. Its potential anti-cancer activity has been well documented [160], and several studies have reported its effect on CRC cell models by arresting the cell cycle at the G1 and G2/M phases [161,162], through the upregulation of p27kip1 [163], cyclin A, cyclin B, and CDK2 [164] and the downregulation of CDK1 [165] and SKP2 [163] protein expression. Additionally, sulforaphane treatment triggered the apoptotic mechanism by increasing ROS levels through glutathione depletion, increasing Ca+ levels, and reducing MMP [161]. This natural compound also activated cytochrome c, DR4, DR5, TRAIL, and caspase-3, -4, -8 and 9 with the consequent upregulation of pro-apoptotic proteins such as Bid and Bax and the downregulation of anti-apoptotic proteins such as Bcl-2, Mcl-1, and XIAP [166,167]. In Figure 5, we have summarized the main molecular mechanisms involved in CRC progression that are controlled by different bioactive compounds.

## 9. Polyphenol Intake to Prevent CRC in Elderly Population: Clinical Studies

Recent statistics show that by 2030, one in six people worldwide will be aged 60 or over, a trend that will see the 60+ population increase from 1 billion in 2020 to 1.4 billion. By the year 2050, the number of people aged 60 and over will increase to 2.1 billion. The 80+ population is projected to triple between 2020 and 2050, to a total of 426 million “https://www.who.int/news-room/fact-sheets/detail/ageing-and-health (accessed on 9 December 2024)”. Aging is complex and diverse and shaped by genetic, environmental, and lifestyle influences [168]. In 2023, C. López-Otín and his team expanded the initial 9 hallmarks of aging to 12, providing a more comprehensive framework for anti-aging studies. Indeed, the most effective geroprotectants focus on these hallmarks, including genomic instability, telomere shortening, epigenetic changes, loss of proteostasis, impaired macroautophagy, nutrient sensing deregulation, mitochondrial dysfunction, cellular senescence, stem cells depletion, altered intercellular communication, persistent inflammation, and dysbiosis [169]. Polyphenols, a major category of phytochemicals present in foods from plants, are of increasing interest for their effects on aging. Several studies have shown that polyphenols have a variety of biological activities, such as anti-inflammatory properties, enhancement of cellular repair, and antioxidant capabilities, thereby reducing the likelihood of age-related diseases such as heart disease, neurodegenerative disorders, and cancer [170,171,172].

In particular, several studies were conducted on older people to evaluate the benefit of polyphenols in counteracting CRC (Table 1). A parallel, open-label, randomized, controlled intervention trial was conducted in thirty-two adult participants, aged 52 to 75 years, identified as being at risk of CRC due to their age, to assess the effect of selenium and EGCG supplementation. EGCG was taken in the form of 200 mg green tea extract (GTE) capsules. The research demonstrated that GTE significantly reduced the levels of rectal DNA methyltransferase mRNA (DNMT1) and NF-κB, both of which play a key role in CRC oncogenesis [173]. As described in detail above, the antitumor potential of resveratrol was documented by investigating its efficacy, safety, and pharmacokinetics in several studies. A two-arm parallel, nonrandomized, placebo-controlled, blinded analysis was conducted on elderly patients with resectable CRC, in order to assess the anticarcinogenic effects of resveratrol. Daily consumption of resveratrol (capsules) at doses of 0.5 g or 1 g for 8 days was sufficient to induce anticarcinogenic effects [174]. Despite the well-established clinical benefits of resveratrol, its therapeutic use is limited due to its quick metabolism and low bioavailability [175]. Curcumin, the most studied phytochemical in both preclinical and clinical research, was evaluated for its efficacy as an anti-inflammatory substance and its potential in the prevention, treatment, and management of various cancer types, including CRC. A recent study investigated the effects of curcumin on older patients with CRC. A total of 126 selected patients were randomly assigned into two groups: the curcumin and the control groups. Patients in the curcumin group were administered 360 mg (in capsule form) of curcumin three times a day during the preoperative phase. The duration of treatment ranged from 10 to 30 days. This study demonstrated that curcumin intake promoted weight loss, reduced serum TNF-α levels, increased apoptosis in tumor cells, boosted p53 proteins, and also affected apoptosis-related Bax and Bcl-2 proteins [176]. Other clinical trials were conducted using curcumin alone or in combination with anthocyanins with very promising results. A randomized, double-blind, placebo-controlled, phase II trial in a small cohort of elderly patients with colorectal adenomatous polyps was conducted to assess the effects of anthocyanin and curcumin or placebo for 4–6 weeks prior to polypectomy. The consumption of natural compounds was found to result in a potentially beneficial adjustment of NF-κB and Ki-67 in colorectal adenomas, suggesting that both inflammation and proliferation could be suppressed by this bioactive combination [177]. Given the role of NF-κB in cancer progression, the inhibition of the NF-κB signaling pathway may represent an important tool in cancer prevention. Another, phase II, randomized, double-blind, placebo-controlled, pre-surgical study was conducted on 35 adult patients with colorectal adenomatous polyps to investigate the biological effects of curcumin and anthocyanins on circulating biomarkers related to inflammation and metabolism. A significant increase in IL-6 was observed at the end of treatment in individuals with high-grade dysplasia. Treatment with both anthocyanins and curcumin did not directly alter circulating inflammatory and metabolism biomarkers but suggested their complex modulation in colon carcinogenesis condition [178]. A clinical trial investigated the safety and tolerability of genistein in combination with a chemotherapy drug in metastatic CRC, in a limited number of patients treated with FOLFOX or FOLFOX-bevacizumab. The results showed that the treatment was safe and well tolerated, with significant effectiveness [179]. Moreover, pomegranate extract was shown to be extensively metabolized by the human gut microbiota due to its content of ellagitannins and ellagic acid, and may regulate the expression of inflammatory genes during cancer, thereby exerting important anti-inflammatory and anti-cancer effects [180].

## 10. Conclusions and Future Perspectives

The discovery of new phytochemicals capable of preventing or treating various types of cancer is one of the key issues in the field of nutraceuticals. Although the onset of CRC is still unknown, it seems to be closely correlated to dietary factors: for this reason, the search for novel natural compounds that are not toxic and that can be used in combination with others therapies to improve the response of cancer cells to chemotherapeutic mediators is imperative. Both in vitro and in vivo models were selected for their ability to respond rapidly, display multistage carcinogenesis expression, tissue/cell specificity, hormone responsiveness, invasiveness, tumor growth modulation, histological types, and specific relevance to the majority of human cancers.

Our review highlighted the latest scientific evidence on the use of phenolic compounds to counteract the development of CRC, with a focus on the adult population. Although the studies reviewed have provided encouraging results, several issues need to be considered when approaching end users. The doses of phytochemicals administered in experimental systems were generally higher than their normal dietary exposure, also considering their bioavailability and toxicity issues, which could probably affect the outcome in clinical trials. In fact, several clinical trials have found low undetectable levels of polyphenols in the blood after oral administration [174,181]. Encapsulation of the compound by nano formulation and chemical modification could address this criticality. Translating findings from animal models to humans can be challenging because of various factors, including host and lifestyle factors, complexity of exposure, metabolic competence, and drug treatments, contributing to a more complex system. This highlights the importance of defining appropriate doses to be administered in order to assess potential functional effects associated with anti-CRC treatments, commonly used in clinical practice. Subsequently, it will be crucial to evaluate the accuracy of the results, including safety and efficacy, in clinical trials. As shown in several of the previously mentioned in vitro and human studies, a precise mixture of selected polyphenols can produce distinctive anti-CRC effects. Clear identification of the maximum tolerable dose of a phytochemical is critical to understanding its therapeutic effectiveness, whether used alone or in combination with another phytochemical or drugs. Regardless, the recognition that the beneficial effects of polyphenols affect almost all the proposed hallmarks of aging in both cellular models and humans may help us to understand the molecular foundation of health progression, reduce the risk of age-related diseases, and increase longevity. Further investigation into this intriguing subject is certainly warranted.

## Figures and Tables

**Table 1:** Polyphenol intake for CRC prevention and treatment in elderly population: evidence in clinical studies.

Polyphenols	Study Subject	Dose and Duration	Main Outcome	References
EGCG	32 adult patients with high risk for CRC	200 mg for 6 weeks	- ↓ DNMT1 - ↓ NF-κB	[173]
Resveratrol	20 adult patients with resectable CRC	0.5 g or 1 g for 8 days	- ↓ tumor cell proliferation by 5% (*p* = 0.05)	[174]
Curcumin	126 adult patients with CRC	360 mg for 10–30 days	- ↑ weight loss - ↓ serum TNF-α levels - ↑ apoptosis in tumor cells - ↑ p53 proteins - affecting apoptosis-related Bax and Bcl-2 proteins	[176]
Curcumin + Anthocyanin	45 adult patients with colorectal adenomatous polyps	500 mg (Curcumin) and 500 mg (anthocyanin) for 4–6 weeks	- ↓ NF-κB immunohistochemistry (IHC) - ↓ Ki-67	[177]
Curcumin + Anthocyanin	35 adult patients with adenomatous polyps	500 mg (Curcumin) and 500 mg (anthocyanin) for 4–6 weeks	- ↑ IL-6 - Modulation in colon carcinogenesis	[178]
Genistein + FOLFOX	13 adult patients with metastatic CRC	Genistein combined with FOLFOX 60 mg/day orally for 7 days every 2 weeks	- Treatment is safe and well tolerated, with significant effectiveness	[179]
Ellagitannins + Ellagic acid	35 adult patients with CRC	900 mg for 5–35 days	- ↑ MYC, CD44, CDKN1A, CTNNB1 - ↓ CASP3	[180]

## Data Availability

No new data were created or analyzed in this study.

## Abbreviations

5-fluorouracil (5-FU), 5-hydroxyheicosatetraenoic acid (5-HETE), Aberrant crypt foci (ACF), Activating protein-1 (AP-1), Age-standardized incidence rate (ASR), Antigen-Presenting Cell (APC), B-Raf proto-oncogene (BRAF), Cell Division Cycle 25C (CDC25C), Chemokine receptor type 4 (CXCR4), Chromosomal instability (CIN), Cleavage poly-ADP-ribose polymerases (cPARP), Colorectal cancer (CRC), CpG island hypermethylation (CIMP), Cyclin-dependent kinase 2 (CDK2), Cyclin-dependent kinase 4 (CDK4), Cyclooxygenase-2 (COX-2), DNA damage-inducible gene 45α (GADD45α), DNA methyltransferase 1 (DNMT1), DNA Methyltransferase 3 Beta (DNMT3B), Epidermal growth factor receptor 3 (ERBB3), Epigallocatechin-3-gallate (EGCG), Familial adenomatous polyposis (FAP), Fas-ligand (FasL), Gallic acid (GA), Green tea extract (GTE), Heat Shock Protein 70 (HSP70), Histone deacetylase 1 (HDAC1), Hypoxia inducible factor 1 subunit alpha (HIF1A), Hypoxia inducible factor 1 subunit alpha (HIF-1A), Inflammatory bowel diseases (IBD), Inhibitor of apoptosis (cIAP-2), Insulin-like growth factor-I receptor (IGF-IR), Interleuchin-6 (IL-6), Kirsten rat sarcoma virus (KRAS), Loss of Heterozygosity (LOH), Lysosomal associated membrane protein 1 (LAMP1), Matrix metalloproteinase-2 (MMP2), Matrix metalloproteinase-9 (MMP9), Microsatellite instability (MSI), Microtubule-associated protein light chain 3 (LC3), Mismatch repair (MMR), Mothers against decapentaplegic (MAD), MutL homolog 1 (MLH1), MutS homolog 2 (MSH2), MutS homolog 6 (MSH6), Nuclear Factor kappa B (NFkB), p38 mitogen-activated protein kinase (p38 MAPK), Phosphatidylinositol 3-kinase (PI3K)/protein kinase B (PKB/AKT), Phospho serine-threonine protein kinase family (p-AKT), Phospho-c-Jun N-terminal kinase1/2 (p-JNK1/2), Phosphorylated extracellular signal-regulated kinase 1 and 2 (p-ERK1/2), Pro-apoptotic protein Bcl-2-associated X protein (BAX), Pro-cancerogenic eicosanoids prostaglandin E2 (PGE2), Reactive oxygen species (ROS), Serine/threonine-protein kinase 2 (Chk2), Signal transducer and activator of transcription 1 (STAT1), Synthesized quercetin 5,3-dihydroxy-3,7,4-triethoxyflavone (TEF), Toll-like receptor 4 (TLR4), Transcription factor EB (TFEB), Transforming growth factor beta (TGFβ), Transforming growth factor beta receptor 2 (TGFBR2), Tumor Necrosis Factor a (TNF-a), Tumor protein p53 (TP53), Vascular Endothelial Growth Factor (VEGF), X-linked inhibitor of apoptosis protein (XIAP).
